# From Proteomic Signatures to Candidate Endotypes in Obesity, Type 2 Diabetes, MASLD/MASH, and MetALD: Study Designs, Bioinformatics, and Biostatistical Strategies

**DOI:** 10.3390/proteomes14030049

**Published:** 2026-09-20

**Authors:** Zhennan Wu, Sachin Anil Ghag, Md Hasan Imam Shihab, Aatman Pushkarkumar Vasoya, Vinamrata Sharma, Jacob Patton Hickman, Yijie Wang, Xiaoqing Huang, Menghao Huang

**Affiliations:** 1 Department of Computer Science, Indiana University Bloomington, 700 N Woodlawn Avenue, Bloomington, IN 47408, USA; zwu1@iu.edu (Z.W.); mshihab@iu.edu (M.H.I.S.); avasoya@iu.edu (A.P.V.); vinshar@iu.edu (V.S.); yijwang@iu.edu (Y.W.); 2 Department of Biochemistry, Molecular Biology & Pharmacology, Indiana University School of Medicine, Indianapolis, IN 46202, USA; ghags@iu.edu; 3 Department of Biostatistics & Health Data Science, Indiana University School of Medicine, 410 W. 10th Street, HITS 3000, Indianapolis, IN 46202, USA; pathickm@iu.edu; 4 Center for Diabetes and Metabolic Diseases, Indiana University School of Medicine, Indianapolis, IN 46202, USA; 5 Melvin & Bren Simon Comprehensive Cancer Center, Indiana University School of Medicine, Indianapolis, IN 46202, USA

**Keywords:** proteomics, proteoforms, endotyping, obesity, type 2 diabetes, MASLD, MASH, MetALD, bioinformatics, biostatistics, multi-omics

## Abstract

Obesity, type 2 diabetes (T2D), metabolic dysfunction-associated steatotic liver disease (MASLD), metabolic dysfunction-associated steatohepatitis (MASH), and dual-etiology metabolic dysfunction-associated alcohol-related liver disease (MetALD) form an overlapping metabolic dysfunction spectrum, rather than a single linear disease sequence. Proteomics offers a functional readout of this spectrum by measuring proteins, proteoforms, and protein species involved in tissue injury, inflammation, metabolic stress, and inter-organ communication. This review asks how proteomic data can support mechanism-based stratification, rather than simply generate disease-associated signatures. We summarize advances in circulating and tissue-based proteomics across obesity, T2D, MASLD/MASH, and MetALD, highlighting shared and disease-specific pathways such as mitochondrial dysfunction, extracellular matrix remodeling, immune activation, proteostasis stress, and endocrine crosstalk. We emphasize that proteomic clusters should be considered candidate endotypes only when they are reproducible, mechanistically coherent, linked to tissue or causal evidence, and clinically informative. We also evaluate bioinformatics and biostatistical strategies needed for reliable interpretation, including preprocessing, missing-data handling, normalization, longitudinal modeling, multi-omics integration, protein quantitative trait locus (pQTL) analysis, colocalization, and Mendelian randomization. Finally, we discuss how proteoforms, post-translational modifications (PTMs), and platform-dependent proteome complexity shape interpretation. Together, these concepts provide practical guidance for moving from proteomic signatures to candidate endotypes and for prioritizing clinically useful biomarkers and therapeutic targets.

## 1. Introduction

Obesity, type 2 diabetes (T2D), and metabolic dysfunction-associated steatotic liver disease (MASLD) are often studied separately, yet they share systemic metabolic dysfunction [1,2,3]. Adipose remodeling, insulin resistance, ectopic lipid deposition, chronic inflammation, mitochondrial stress, and tissue injury link these conditions across organs [4,5,6,7,8]. In this landscape, liver disease is not an isolated endpoint: obesity and insulin resistance can contribute to steatosis, metabolic dysfunction-associated steatohepatitis (MASH), fibrosis, and, in some patients, mixed metabolic and alcohol-related liver injury [2,9,10]. This connected view provides a stronger framework for interpreting molecular data and identifying biologically meaningful subgroups [10,11]. Some individuals with obesity retain relatively preserved metabolic health and low ectopic liver fat, whereas others develop insulin resistance, T2D, hepatic steatosis, steatohepatitis, fibrosis, or mixed metabolic–alcohol-related liver injury. MASLD may also precede, coexist with, or follow T2D, and progression from steatosis to metabolic dysfunction-associated steatohepatitis (MASH) or fibrosis occurs only in a subset of patients [12,13]. We therefore use the term “metabolic dysfunction spectrum” to describe overlapping risk states and shared mechanisms, not a fixed chronological sequence.

Recent nomenclature changes reinforce this mechanism-based view. MASLD, MASH, and metabolic dysfunction-associated alcohol-related liver disease (MetALD) align liver disease labels more closely with metabolic and mixed-etiology biology [4,10]. However, clinical classification has advanced more rapidly than molecular stratification [11]. Patients with the same diagnosis may differ in inflammatory burden, lipid handling, fibrosis risk, mitochondrial dysfunction, and therapeutic response [14,15]. These differences motivate a shift from broad phenotypes toward candidate endotypes defined by disease-driving mechanisms [11,16].

Proteomics is well suited to support endotype discovery. Genomics captures inherited risk, and transcriptomics captures gene-transcription programs, whereas the proteome more directly reflects functional biology [17,18]. Proteins execute cellular processes and integrate genetic, environmental, secretory, complex formation, turnover, and post-translational regulatory layers that cannot be fully inferred from ribonucleic acid (RNA) [17,19]. In metabolic disease, protein abundance and modification also reflect nutrient exposure, hormonal state, inflammation, inter-organ crosstalk, and temporal progression [20,21]. Profiling plasma, tissue, extracellular vesicles (EVs), and spatial samples can therefore connect mechanisms to clinically relevant phenotypes more directly than any single omics layer alone [22,23].

Proteomic evidence in metabolic disease remains fragmented. Studies have identified adipose remodeling, insulin resistance, hepatic inflammation, extracellular matrix (ECM) deposition, mitochondrial dysfunction, and circulating biomarker candidates [23,24]. Yet many analyses are disease-specific, cross-sectional, modest in scale, or limited to a single biospecimen type [18,20]. Results also depend on the platform, sample source, and statistical controls for age, sex, adiposity, medication use, alcohol exposure, and disease stage [18,25]. These differences make it difficult to compare studies, separate shared from disease-specific signatures, and distinguish mechanistic proteins from associative markers [21,23].

A cross-disease framework can move the field beyond descriptive lists of proteins toward proteome-informed endotyping [11,21]. Key questions are which proteomic patterns recur across obesity, T2D, and MASH; which define divergent trajectories; and which analytical strategies identify robust and clinically meaningful subgroups [20,23]? Bioinformatics and biostatistics are central to these questions [26,27]. Preprocessing, quality control (QC), missing-data handling, normalization, batch correction, covariate adjustment, longitudinal modeling, network analysis, multi-omics integration, and causal inference all shape the biological conclusions drawn from proteomic data [28,29,30,31].

This review focuses on how proteomic data can be used responsibly to stratify patients across obesity, T2D, MASLD/MASH, and MetALD. We emphasize three questions: First, which biological processes recur across these conditions, and which are organ- or stage-specific? Second, what study design choices are required for reliable population-based or tissue-based proteomics? Third, what computational and validation standards are needed before a proteomic pattern can be interpreted as a candidate endotype? Rather than simply cataloging proteomic studies, we use this spectrum-based framework to identify recurring gaps in platform choice, tissue–plasma interpretation, covariate modeling, longitudinal design, biomarker validation, proteoform awareness, and causal inference. We summarize major proteomic platforms, review findings across these disorders, and provide practical computational recommendations to connect descriptive proteomics with clinically actionable disease stratification.

## 2. Proteomic Platforms and Study Design

### 2.1. Scope, Terminology, and Literature Search Approach

For this review, we distinguish proteomic signatures from mechanism-based endotypes. A phenotype is an observable clinical or biological state, whereas a clinical subtype is defined primarily by clinical characteristics, outcomes, or risk profiles. A proteomic signature refers to a set of proteins or proteoforms associated with a disease state, outcome, or treatment response; such a signature should not, by itself, be considered an endotype. Consistent with the established use of the term, an endotype denotes a subgroup defined by a distinct pathobiological mechanism [32]. We therefore use candidate endotype for a reproducible proteomic subgroup supported by biological plausibility and additional evidence, such as tissue concordance, longitudinal behavior, genetic or causal support, external replication, or differential treatment response, but not yet sufficiently validated for routine clinical use. We reserve validated endotype for a subgroup whose underlying mechanism and clinical relevance have been reproduced across independent studies.

The term metabolic dysfunction spectrum is used to describe the overlapping and bidirectional relationships among adiposity, insulin resistance, T2D, MASLD/MASH, and MetALD. This term does not imply a fixed sequence of disease progression. Metabolically healthier obesity can occur; MASLD may precede, coexist with, or follow T2D, and only a subset of patients progresses from steatosis to MASH or advanced fibrosis [10,12,33,34].

For this narrative review, we conducted targeted searches of PubMed and Web of Science through August 2026 and supplemented these searches by screening the reference lists of relevant articles. Search concepts combined proteomics, proteoforms, or protein abundance with obesity, adipose tissue, insulin resistance, T2D, MASLD, MASH, MetALD, biomarker, endotype, longitudinal analysis, protein quantitative trait locus, colocalization, Mendelian randomization (MR), and multi-omics. We prioritized human and translational studies, key methodological studies, and reports linking proteomic measurements to tissue biology, disease progression, treatment response, or external validation. Because this review was designed as a narrative synthesis, rather than a systematic review or meta-analysis, studies were selected according to their relevance to the review questions and their methodological or translational contributions, rather than through a formal systematic review protocol [35].

Selection of the specimen context and proteomic measurement strategy determines which biological and translational questions can be answered across obesity, T2D, MASLD/MASH, and MetALD. Proteomic approaches differ in sample requirements, depth, throughput, tissue specificity, and interpretability, so the approach should be chosen according to the intended inference, rather than as a purely technical preference [36]. Cross-platform studies further show that assay choice influences both protein detection and disease associations [25]. Because each approach captures different aspects of the metabolic disease spectrum, platform selection should be guided by the biological question, sample type, cohort scale, and intended translational use. Table 1 summarizes the major platform choices and design considerations relevant to proteome-informed endotyping. In this review, “platform” is used broadly, while Table 1 distinguishes specimen context from analytical strategy and emphasizes limitations and validation needs.

### 2.2. Plasma and Tissue Proteomics Provide Complementary Views of Metabolic Disease

Plasma/serum and tissue proteomics provide distinct but complementary information for endotype discovery. Plasma supports scalable biomarker discovery, longitudinal monitoring of systemic insulin resistance, and noninvasive risk stratification (e.g., differentiating isolated steatosis from active MASH), but interpretation is limited by dynamic range and uncertain tissue origin [36,37,38,39]. Conversely, direct tissue proteomics—specifically of liver biopsies or visceral adipose tissue—resolves local pathogenic drivers, such as hepatic lipotoxicity, stellate cell activation, and adipocyte dysfunction. Paired tissue–plasma designs are therefore critical; they allow statistical models to map circulating proteins back to active tissue-level biology, effectively translating local histological damage into accessible, noninvasive endotypes [40,41,42,43,44,45,46]. Orthogonal confirmation may combine targeted MS or an independent affinity assay, replication in an external cohort, tissue–plasma concordance, and longitudinal or genetic support.

### 2.3. Mass Spectrometry (MS)-Based Discovery Proteomics

MS-based discovery proteomics remains central to broad protein discovery because it provides sequence-specific identification and flexible quantification in plasma and tissue [36]. Data-dependent acquisition (DDA) supports deep exploratory profiling of localized lipotoxic or fibrotic liver injury and spectral library generation, whereas data-independent acquisition (DIA) improves completeness and reproducibility across large, heterogeneous cohorts and provides the quantitative completeness necessary for population-level studies [47,48,49,50,51,52]. DDA and DIA are complementary, rather than interchangeable; acquisition design, software, spectral library strategy, and missingness should be reported, and high-priority candidates should be confirmed by targeted MS.

### 2.4. Affinity-Based Proteomics in Large Metabolic Cohorts

Affinity-based platforms are well suited to large plasma cohorts because Olink and SomaScan assays enable high-throughput profiling of circulating readouts of hepatic stress and systemic inflammation from small sample volumes [53,54,55]. They are powerful for association discovery, subtype analysis, and proteogenomics, but partial cross-platform concordance means that high-priority findings, such as those meant to distinguish aggressive MASH or MetALD from isolated steatosis, should be confirmed orthogonally [25,53,55,56,57,58,70]. Affinity signals may also be influenced by antibody–epitope or aptamer-binding effects; agreement with MS, an independent affinity reagent, or tissue/genetic evidence strengthens interpretation [70].

### 2.5. Targeted Proteomics for Verification and Translation

Targeted proteomics bridges discovery and validation by quantifying predefined panels with selected reaction monitoring (SRM), multiple reaction monitoring (MRM), or parallel reaction monitoring (PRM). It is particularly useful for verifying biomarker candidates related to fibrosis, inflammation, adipose dysfunction, or insulin resistance [36,40,59]. These methods require prior target selection, suitable proteotypic peptides, and analytical standards.

### 2.6. Post-Translational Modification (PTM)-Focused Proteomics Adds Mechanistic Resolution

PTM-focused proteomics adds mechanistic resolution by capturing signaling and regulatory states that may not manifest as changes in total abundance. Phosphoproteomics is especially relevant to insulin signaling, nutrient sensing, inflammation, and steatohepatitis biology [5,60]. Interpretation requires enrichment, reliable site localization, and sufficient coverage; a PTM change should not be assumed to reflect a change in total protein abundance.

### 2.7. Secretome and EV Proteomics for Inter-Organ Crosstalk

Secretome and EV proteomics address inter-organ communication by identifying released proteins and vesicle cargo from adipose tissue, liver, muscle, pancreas, and circulation [61,62,63,64]. Because EV profiles depend on isolation and preparation, these approaches are strongest when paired with standardized collection, isolation, purity assessment, and orthogonal validation [65,66]. Confirmation with an independent isolation method or targeted assay is particularly important because preparation choices can alter detected cargo.

### 2.8. Single-Cell and Spatial Proteomic Approaches: Current Capabilities and Limitations

Single-cell and spatial proteomics can refine endotyping by resolving cell states, tissue niches, and zonated pathology that are masked in bulk assays. These tools remain less mature for large biomarker studies, but they are increasingly valuable for mechanistic refinement [67,68,69]. Current limitations include lower throughput, incomplete proteome coverage, and the need to validate cell-state findings in independent tissues or orthogonal assays.

## 3. Obesity and Adipose Dysfunction

### 3.1. Proteomic Themes in Adipose Dysfunction

Obesity is a major risk factor within the metabolic dysfunction spectrum that includes T2D and MASLD. Adipose tissue is not an inert lipid depot, but an endocrine and immune-active organ whose pathological expansion produces reproducible proteomic shifts [71]. Obesity proteomics consistently implicates ECM remodeling, fibrotic cross-linking, oxidative stress, and pro-coagulant pathways [72,73,74]. Redox-focused studies further point to mitochondrial exhaustion, endoplasmic reticulum (ER) stress, impaired mitochondrial protein import, and the accumulation of oxidized proteins [75]. These changes disturb the adipokine secretome and link local adipose dysfunction to systemic low-grade inflammation.

### 3.2. Biological Heterogeneity and Tissue–Plasma Discordance

Obesity proteomics is complicated by marked clinical heterogeneity, particularly the distinction between metabolically healthy obesity (MHO) and metabolically unhealthy obesity (MUO). MHO is associated with preserved insulin sensitivity and more benign hyperplastic expansion, whereas MUO is linked to hypertrophic adipocyte stress, fibrosis, and ectopic lipid deposition [76]. Targeted plasma proteomics can distinguish these phenotypes through immune, growth-factor, and chemoattractant signatures before overt metabolic disease appears [77].

Proteomic discovery should therefore not rely on body mass index (BMI) alone. Sensitive endotyping is needed to capture divergent molecular trajectories and to address tissue–plasma discordance. Adipose tissue proteomics can reveal local macrophage infiltration and ECM stiffening, whereas circulating proteins reflect a composite signal shaped by dilution, secretion dynamics, and renal or hepatic clearance. As a result, plasma profiles often only partly recapitulate the molecular state of adipose tissue.

### 3.3. Obesity-Specific Study Design Considerations

Obesity heterogeneity and tissue–plasma discordance create major biostatistical challenges. Proteomic profiles are strongly influenced by age, sex, diet, physical activity, and medications such as glucagon-like peptide-1 (GLP-1) receptor agonists and statins [74,78,79,80,81,82]. Because obesity is a common upstream risk state across much of the metabolic dysfunction spectrum, inadequate adjustment can yield overfit biomarkers and associations that fail to replicate.

Obesity proteomics, therefore, needs more than simple case–control designs. Matched cohorts, unsupervised clustering of MHO/MUO endotypes, and covariate-adjusted differential analysis are essential. These issues also foreshadow the missingness, confounding, and feature-selection challenges that become more complex in T2D and MASLD/MASH. General preprocessing, missingness, normalization, batch, and validation issues shared across all disorders are addressed in Section 7.

## 4. Insulin Resistance and T2D

Adipose dysfunction, insulin resistance, and T2D represent a major node in the metabolic dysfunction spectrum, linking adipose remodeling to systemic metabolic stress and liver disease biology [3,10,83,84,85]. T2D is heterogeneous, arising from variable degrees of insulin resistance, impaired insulin secretion, adiposity, inflammation, and tissue-specific dysfunction [83,84,86]. Peripheral and hepatic insulin resistance increase insulin demand, and diabetes develops when beta cells cannot compensate [83,87]. Chronic nutrient excess, ectopic lipid deposition, inflammation, oxidative stress, and ER stress further link obesity to MASLD/MASH progression [10,87,88,89,90]. Clinically similar patients can differ in age at onset, beta-cell reserve, comorbidities, complications, and treatment response [84,86,91]. These differences reflect variable contributions from peripheral and hepatic insulin resistance, beta-cell dysfunction, adipose inflammation, and cardiometabolic risk [57,83,84]. Within the spectrum, T2D is a metabolically active state in which obesity, dyslipidemia, steatosis, inflammation, and mixed metabolic–alcohol exposures can amplify injury [10,87,88]. Proteomics is useful because circulating and tissue proteins may capture inter-organ biology not represented by glucose-centered measures [37,57,92]. This heterogeneity has motivated clinical subclassification [86,91]. Proteomics supports it by identifying subtype-associated signatures linked to complement activation, insulin signaling, adipokines, and inflammatory tone [57,92]. Yet plasma signatures should be interpreted with caution, as they may offer only modest predictive value beyond strong clinical models [37,93]. Thus, prediction asks whether proteins improve risk scores, whereas biological stratification asks whether they reveal mechanisms or endotypes [37,91,92]. Current T2D proteomics is strongest as a tool for biologically informed stratification and target discovery, not as a replacement for routine models [37,57,92]. Clinical use will require benchmarking, external validation, standardized assays, diverse cohorts, and longitudinal outcome data [37,92,94].

### 4.1. Proteome-Informed Subtype/Endotype Discovery

Proteome-informed endotyping extends clinical subtyping: clinical clusters separate broad phenotypes, while proteins assess whether those phenotypes reflect distinct biology [57,86,91]. Subtype studies show that circulating molecular signatures can distinguish T2D subgroups. Recent large-scale proteomic analyses have further identified inflammation- and lipid-related molecular subgroups before overt T2D, supporting the potential of proteomics to refine early metabolic risk stratification [95]. Reported patterns include complement activation and reduced 1,5-anhydroglucitol in severe insulin-deficient diabetes, impaired insulin signaling in severe insulin-resistant diabetes, and higher levels of leptin and fatty acid-binding proteins in mild obesity-related diabetes [57]. Multi-omics studies further link T2D heterogeneity to islet function, adipose biology, liver metabolism, and genetically informed cardiometabolic pathways [57,84,96]. However, subtype frameworks are not yet ready for routine clinical use because reproducibility, transferability, outcome association, and treatment utility remain uneven [91,94]. A systematic review found that complex stratification methods yielded more reproducible outcome-associated subtypes than many simpler approaches [91]. Therefore, proteome-informed endotyping should be presented as promising but still developing [91,94]. More precise subclassification could explain differences in complications and treatment response, and plasma-detectable signals make proteomics attractive for biomarker panels [57,86,91]. Translation will require validated biomarkers, scalable assays, diverse cohorts, medication adjustment, and evidence that subtype assignment changes clinical decisions [92,94]. Future studies should integrate proteomic, clinical, genetic, metabolomic, and longitudinal data to link circulating biomarkers to etiology, complications, therapy response, and liver or cardiovascular outcomes [57,84,94].

### 4.2. Plasma Proteomic Signatures Beyond Clinical Scores

Plasma proteomics offers an accessible readout of insulin resistance, inflammation, adipose dysfunction, vascular risk, and tissue injury in T2D [37,92,93]. Protein signatures can improve the estimation of directly measured insulin sensitivity, and smaller, stable protein sets may retain this benefit across cohorts [93]. For incident T2D prediction, however, gains over strong clinical baselines are often modest. In the United Kingdom (UK) Biobank, a protein-only least absolute shrinkage and selection operator (LASSO) model performed similarly to QDiabetes, and adding proteins to clinical/genetic predictors yielded only limited additional improvement [37]. Proteomics should therefore not be judged solely by population-level prediction metrics [37,92]. Its value may be greater for biological stratification, subgroup discovery, treatment response characterization, and target identification [57,92,93]. Plasma signatures are best viewed as complementary tools for stratification and risk refinement, not as stand-alone replacements for clinical scores [37,92]. Progress depends on benchmarking, smaller, stable panels, platform harmonization, and validation across sites, ancestries, and disease stages [37,92,93,94].

### 4.3. Implications for Clinical Translation and Study Design

For clinical translation, T2D proteomic endotypes must be reproducible, externally validated, linked to meaningful outcomes, and useful for prevention or treatment decisions [91,94]. This requires scalable subtype-informing biomarkers and cohorts that reflect real-world diversity in ancestry, sex, age, adiposity, medications, and liver disease. Larger longitudinal cohorts, shared data, harmonized covariates, and cross-study benchmarking are needed to separate robust biology from setting-specific findings [92,94]. Platform standardization is also essential because affinity and MS methods differ in target recognition, quantitative performance, and biomarker portability [25,92]. Models should adjust for age, sex, adiposity, glycemia, medication use, kidney function, liver disease status, and cardiometabolic comorbidities [92,94]. MASLD/MASH status should be measured or modeled because liver disease is both a consequence of insulin resistance and a contributor to circulating proteins in T2D [10,88]. Longitudinal sampling is important because insulin resistance, beta-cell stress, medications, adiposity, liver fat, inflammation, and protein abundance change over time [93,94]. Ultimately, utility depends on demonstrating that subtype-informed biomarkers improve real-world diagnosis, prevention, prognosis, treatment selection, or monitoring [91,94]. T2D proteomics should therefore be framed as a bridge from clinical heterogeneity to mechanism-based endotyping across the metabolic dysfunction spectrum, rather than as a replacement for established risk models [37,57,92].

## 5. MASLD/MASH and MetALD: Proteomic Overlap and Divergence

**Figure 1:**
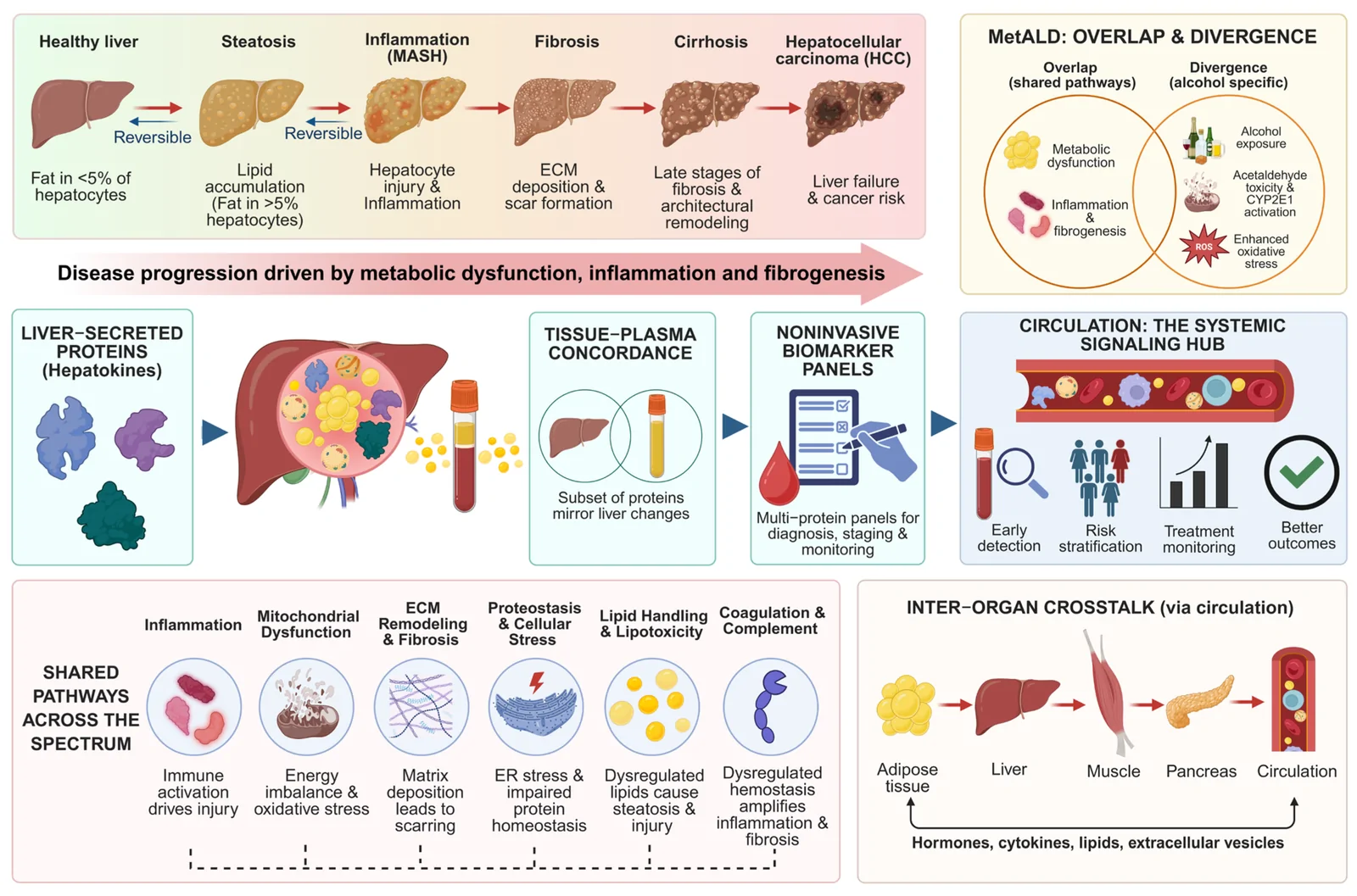
Proteome-informed framework for metabolic liver disease stratification. The schematic positions MASLD/MASH and MetALD within the obesity–T2D–metabolic liver disease axis. It summarizes possible disease trajectories, liver-secreted proteins, tissue–plasma concordance, noninvasive biomarker panels, circulating proteomic signals, shared pathways, and inter-organ crosstalk mediated by hormones, cytokines, lipids, and extracellular vesicles (EVs). Arrows indicate possible paths and shared mechanisms, rather than a required chronological sequence. Created with BioRender.com (https://BioRender.com/emx09n5, accessed on 17 September 2026). Abbreviations: ECM, extracellular matrix; ER, endoplasmic reticulum; CYP2E1, cytochrome P450 family 2 subfamily E member 1; ROS, reactive oxygen species.

MASLD is an umbrella diagnosis requiring hepatic steatosis plus cardiometabolic risk features. MASH refers to the inflammatory and hepatocellular injury subtype. Fibrosis, cirrhosis, and hepatocellular carcinoma are possible outcomes in a subset of patients, with progression driven by lipid accumulation, hepatocellular stress, inflammation, and fibrogenesis [3,41,90,97,98]. Early lipotoxicity and oxidative stress promote hepatocyte injury [50,97], while persistent injury activates hepatic stellate cells and ECM deposition [41]. Because these processes unfold within the broader obesity–T2D–metabolic liver disease spectrum and may overlap with alcohol-related injury in MetALD, an integrated proteomic framework is needed to connect liver disease progression with tissue–plasma concordance, circulating biomarkers, and inter-organ signaling (Figure 1).

### 5.1. Noninvasive Biomarker Panels and Risk Stratification

Early MASLD begins with triglyceride storage in hepatocyte lipid droplets. When storage capacity is exceeded, or lipid composition shifts toward toxic species such as saturated free fatty acids and ceramides, lipotoxicity develops [99]. These lipids damage cellular membranes and organelles, including the ER. ER stress activates the unfolded protein response, including the inositol-requiring enzyme 1 alpha (IRE1A) pathway. Chronic IRE1A activation can promote the release of proinflammatory EVs that recruit and activate immune cells in the liver microenvironment [100].

The following lipidomic observations are included only as complementary mechanistic context and are not treated as proteomic measurements. Lipidomic studies show that MASH differs from simple steatosis through specific remodeling patterns. Reported changes include reduced ether-linked phosphatidylethanolamine, increased ester-linked phosphatidylethanolamine, elevated saturated sphingomyelin species, and correlations between cathepsins and linoleic acid-containing triglycerides, linking lysosomal dysfunction with altered fatty acid metabolism [97]. As an endocrine organ, the liver also secretes hepatokines and other proteins that reflect intrahepatic metabolic and inflammatory states; MS-based studies report extensive remodeling of the liver-secreted proteome in MASH [40,41]. Because not all individuals with obesity progress to MASH, the APASHA model integrates circulating proteins with clinical variables to identify high-risk individuals [42]. This emphasis on noninvasive biomarker panels directly addresses biopsy limitations [40,42]. Secreted factors such as APOF, PCSK9, AFM, S100A6, and AZGP1, combined with glycated hemoglobin (HbA1c), capture lipid metabolism, inflammation, and insulin resistance [42]. Importantly, plasma remodeling is more pronounced in MASH than in simple steatosis, suggesting a transition point at which liver pathology becomes systemically detectable [40,41]. Longitudinal plasma proteomic studies further suggest that disease-associated protein signatures can precede clinically recognized MASLD by many years, supporting their potential utility for early risk stratification and temporal validation [101].

Integrated liver–plasma proteomics shows that only a subset of plasma proteins reflects changes in liver tissue [40,41]. In paired analyses, roughly 30 plasma proteins correlate with their liver counterparts, and fewer consistently track with progression [40]. These concordant proteins are valuable because they link biopsy-based biology to noninvasive assessment [40,41]. Selected plasma markers can distinguish MASH from non-MASH and identify advanced fibrosis [41,42]. Targeted profiling across cohorts has identified a seven-protein signature comprising CASP-8, CCL20, CTSD, SCF, MMP-3, TRAIL, and TWEAK [42]. However, liver–plasma discordance also indicates that circulating signals may reflect extrahepatic biology, underscoring the importance of multi-omics integration for robust biomarker development [40,41].

Protein abundance alone does not capture the full biology of MASLD because proteoforms and PTMs also influence disease onset and progression [102,103,104,105]. PTMs alter protein function, stability, localization, and protein species behavior, and persistent PTM patterns can contribute to chronic liver injury [102,103]. Relevant modifications include phosphorylation, ubiquitination, acetylation, and glycosylation [104,105]. The concept of PTM memory suggests that chronic liver disease can persist through durable proteoform-level modification states [102]. Emerging data indicate that distinct liver cell types maintain such memory through specific PTM pathways [103,105].

### 5.2. MetALD: Overlap and Divergence with MASLD/MASH

Proteomic and metabolomic evidence is discussed here as complementary but non-interchangeable: proteins report effector and signaling states, whereas metabolites primarily report pathway substrates and products. MetALD reflects the frequent coexistence of metabolic dysfunction and alcohol exposure [106,107]. These drivers can synergize to worsen liver injury, inflammation, and fibrosis [106]. Proteomic and metabolomic studies indicate that MetALD is heterogeneous, with some profiles closer to MASLD and others closer to alcohol-associated liver disease [107]. Circulating biomarker approaches can distinguish subtypes and predict long-term outcomes, including cardiovascular events, cirrhosis, and mortality [106,107]. Alcohol-specific mechanisms, including acetaldehyde-induced oxidative stress and CYP2E1 activation, may exacerbate insulin resistance- and lipotoxicity-driven injury [106]. Thus, MetALD shares altered lipid handling and inflammation with MASLD/MASH but diverges through alcohol-related toxicity and oxidative stress [106,107].

Overall, MASLD/MASH and MetALD represent a liver-centered axis driven by metabolic stress, inflammation, and fibrogenesis. Plasma proteomics increasingly captures systemic manifestations of this process, while liver-secreted protein profiling, tissue–plasma concordance analysis, and multi-marker panels are improving noninvasive stratification. MetALD emphasizes the need for biomarkers that account for both metabolic and alcohol-related drivers.

## 6. Shared Pathways Across the Spectrum

The cross-disease argument is based on recurring proteomic modules, rather than a presumed chronological sequence. Across organs, MASLD/MASH and MetALD share pathways that drive disease initiation, progression, and systemic complications. These processes link adipose tissue, liver, skeletal muscle, pancreas, and the circulating proteome [108,109]. As outlined in Figure 1 and summarized in Table 2, these shared mechanisms help explain why plasma proteomics can capture systemic disease activity and support noninvasive endotyping across the metabolic dysfunction spectrum. A candidate endotype is therefore most credible when the same coordinated protein program recurs across cohorts or tissues and is linked to a plausible mechanism or clinical trajectory.

### 6.1. Core Mechanistic Pathways

Inflammation, mitochondrial dysfunction, oxidative stress, ECM remodeling, proteostasis stress, lipotoxicity, and complement/coagulation activation recur across the metabolic dysfunction spectrum. These pathways connect adipose dysfunction with hepatic injury, systemic insulin resistance, fibrosis risk, and circulating biomarker signatures (Table 2).

### 6.2. Inter-Organ Crosstalk and Circulation

Circulation links these tissue programs through adipokines, hepatokines, myokines, pancreatic stress signals, and EV cargo. This system-level view explains why plasma and EV proteomics can support early detection, risk stratification, treatment monitoring, and assessment of disease resolution, while also requiring cautious interpretation because circulating proteins can arise from multiple tissues (Table 2 and Figure 1).

## 7. Bioinformatics, Biostatistics, and Computational Frameworks

Bioinformatics and biostatistics determine whether multi-organ proteomic studies yield reproducible biological findings or technical artifacts. Unlike many other omics workflows, proteomic analysis depends strongly on specimen type, measurement modality, and the molecular unit being quantified. In mass spectrometry (MS)-based studies, spectra are assigned to peptides, which are then used to infer proteins or protein groups; post-translational modification-focused analyses additionally require reliable site localization. Affinity-based assays instead generate reagent-dependent target signals, whose specificity and cross-platform concordance must be evaluated independently. Accordingly, data-dependent acquisition, data-independent acquisition, targeted MS, and affinity-based assays differ in dynamic range, missingness, normalization requirements, quantification units, protein inference, and validation strategies [17,126,127,128].

**Figure 2:**
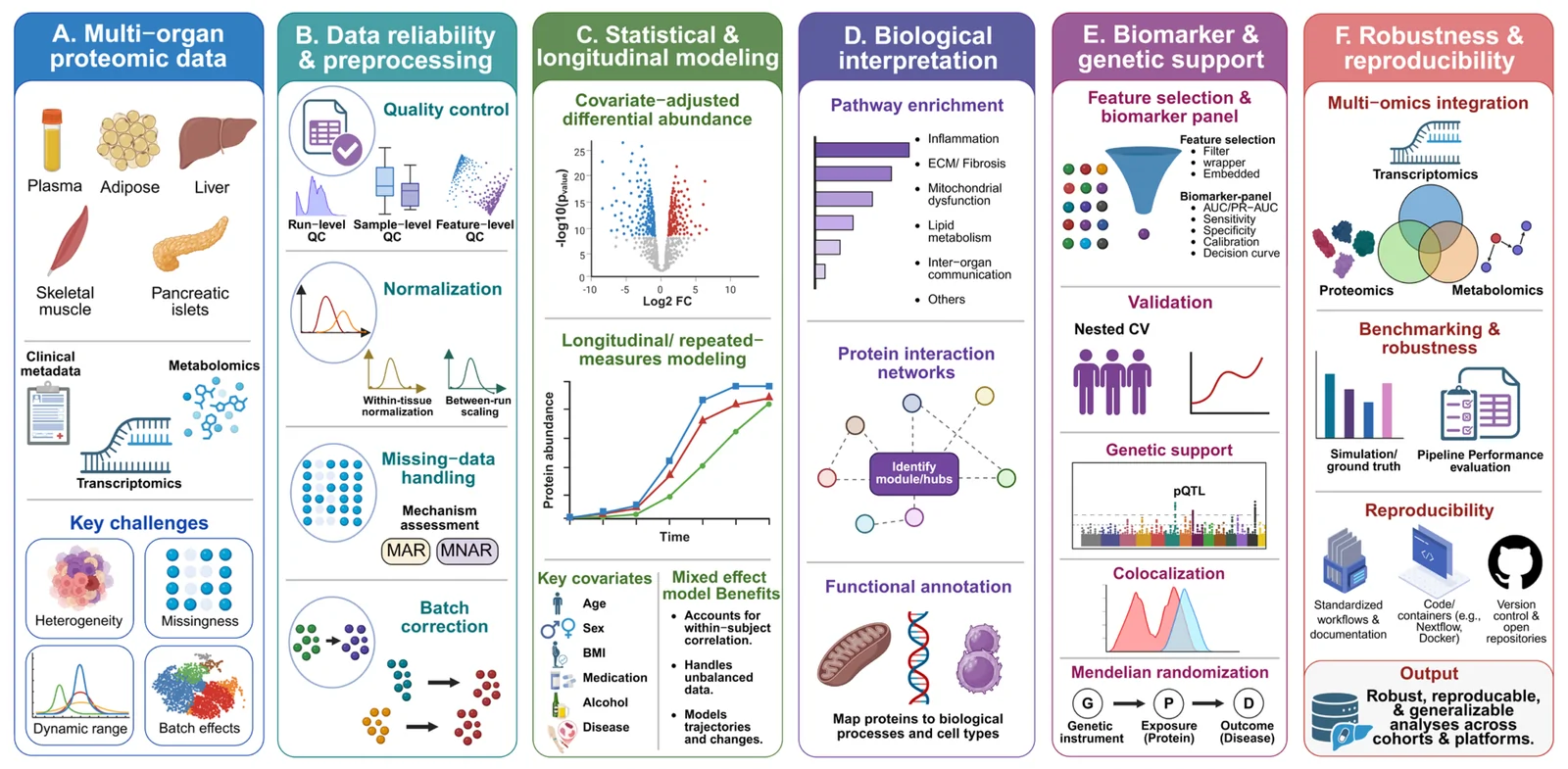
Computational workflow for proteome-informed endotyping. The workflow begins with multi-organ proteomic data and linked clinical or omics metadata (**A**), followed by QC, normalization, missing−data handling, and batch correction (**B**). Differential−abundance and longitudinal models identify disease-, trajectory-, and treatment-associated proteins while accounting for covariates (**C**). Pathway enrichment, protein interaction networks, and functional annotation support interpretation (**D**). Feature selection, validation, protein quantitative trait locus (pQTL) analysis, colocalization, and Mendelian randomization (MR) prioritize candidate biomarkers and therapeutic targets (**E**). Multi-omics integration, benchmarking, standardized workflows, version control, and open repositories strengthen reproducibility and cross-cohort generalizability (**F**). Created with BioRender.com. Abbreviations: BMI, body mass index; MAR, missing at random; MNAR, missing not at random; FC, fold change; ECM, extracellular matrix; CV, cross-validation; AUC, area under the curve; PR-AUC, area under the precision–recall curve.

These distinctions are especially important in obesity, type 2 diabetes, metabolic dysfunction-associated steatotic liver disease/metabolic dysfunction-associated steatohepatitis, and metabolic dysfunction-associated alcohol-related liver disease. Lipid-rich adipose tissue, steatotic or fibrotic liver, hyperglycemia-associated protein modifications, altered hepatic or renal clearance, medication exposure, and alcohol use may affect both analytical measurement and biological interpretation. Analytical choices should therefore be matched to the specimen, platform, missingness structure, batch design, clinical covariates, and intended inference [128,129]. Figure 2 summarizes the analytical workflow, while Table 3 compares the principal tasks and practical recommendations. Section 7.1, Section 7.2, Section 7.3, Section 7.4, Section 7.5, Section 7.6, Section 7.7, Section 7.8 and Section 7.10 address core proteomic analyses, whereas Section 7.9 considers complementary multi-omics integration.

### 7.1. Preprocessing and QC

Preprocessing and QC determine whether observed differences reflect biology or technical variation. They should be tissue- and platform-aware, as the severe lipid accumulation (steatosis) and ECM deposition (fibrosis) in MASLD/MASH livers profoundly alter protein extraction efficiency. Similarly, the hypertrophic and hypoxic environment of obese adipose tissue, combined with the hyperlipidemia and hyperglycemia inherent to T2D, creates severe matrix effects and spontaneous protein glycations [130,131,132]. Discovery DDA/DIA and targeted MRM workflows also differ in signal structure and susceptibility to technical variability, so they should not be processed identically [191,192,193].

A reproducible workflow should track provenance from raw spectra or assay outputs to the protein matrix, including peptide identification, protein inference, quantification, filtering, and software versions. MaxQuant, Perseus, and MSnbase illustrate how structured pipelines can support reproducible processing when assumptions are documented [131,132,194].

QC should be evaluated at run, sample, and feature levels using proteome depth, intensity distributions, principal component analysis (PCA) by tissue and batch, missingness maps, sample correlations, replicate metrics, and contaminant or sparsity filters. Exclusions should rely on concordant evidence across diagnostics and metadata, and reporting should include acquisition mode, batch structure, QC metrics, exclusion criteria, and repository deposition [133,134,195].

### 7.2. Missing-Data Handling

Missingness arises from low-abundance censoring, stochastic precursor selection, peptide detectability, and batch or run instability. These mechanisms can correspond to missing at random (MAR) or missing not at random (MNAR) structures and may be informative in metabolic disease when low-abundance signaling proteins or tissue-restricted proteins are inconsistently detected [30,135]. Benchmarking studies further indicate that imputation performance depends on the structure and missingness characteristics of the dataset, arguing against the routine use of a single default imputation method [196].

Missingness should be quantified by protein, sample, group, and tissue before filtering, imputation, or model-based inference. Dropout plots, prevalence distributions, missingness heatmaps, and sensitivity analyses make the workflow auditable, while methods such as MSqRob and MSstats reduce reliance on default imputation when missingness is substantial or structured [30,135,136,137,138,139,140].

### 7.3. Normalization and Batch Correction

Normalization and batch correction are applied after QC and initial missingness assessment and before downstream modeling. Across the obesity, T2D, and steatotic liver disease spectrum, the fundamental objective is to eliminate technical noise without suppressing the subtle, overlapping biological gradients that define distinct endotypes. This balance becomes challenging when batch aligns with tissue type, disease stage, acquisition site, or processing time [135,141,142].

Normalization should generally be performed within analytically homogeneous tissues or platforms and evaluated using diagnostics such as intensity distributions, sample-centered variability, multivariate structure, and replicate consistency. Batch correction methods such as surrogate variable analysis or ComBat can help when unwanted variation is separable, but overcorrection can remove true tissue or disease signals; therefore, correction strategies should be evaluated using pre- and post-correction diagnostics and sensitivity analyses [130,141,142,143,144,197].

### 7.4. Differential Abundance with Covariate Adjustment

Protein abundance in the context of the obesity–T2D–MASH network is deeply confounded by systemic metabolic dysfunction, overlapping comorbidities, and polypharmacy, making covariate adjustment essential. Factors such as baseline adiposity (BMI or visceral fat mass), glycemic control (glycated hemoglobin [HbA1c]), alcohol intake (critical for resolving MetALD), and concurrent medication use (e.g., metformin, statins, or GLP-1 receptor agonists) can easily generate massive false-positive signatures or mask true protein–disease associations [145,146]. Linear-model frameworks such as limma remain foundational because their design matrices can systematically isolate specific pathological effects, while empirical Bayes moderation stabilizes the high variance typical of diverse clinical cohorts [147,148].

Alternative models should be selected according to the data structure. MSstats models peptide-to-protein variability with mixed-effects frameworks, DEqMS accounts for peptide spectrum-match or peptide count-dependent variance, and 2dFDR integrates confounder adjustment with multiple testing control to improve power in high-dimensional omics [139,152,153,198,199].

Thus, differential abundance analysis should not use a single default model across all metabolic proteomics studies. The strongest framework is the one that matches the platform, missingness, peptide structure, covariate pattern, and validation goal.

### 7.5. Longitudinal and Repeated-Measures Models

Longitudinal proteomic designs are valuable because obesity, T2D, MASLD/MASH, and MetALD evolve through gradual remodeling, shifting inflammation, and treatment-responsive molecular change. Repeated sampling captures within-person trajectories and can distinguish dynamic disease biology from static cross-sectional associations [56,154,155].

Linear mixed-effects models are the practical starting point because they account for correlated repeated samples, unequal visit numbers, subject-specific baselines, and time-varying covariates such as BMI, glycemia, medication exposure, alcohol exposure, and disease stage [56,154,155]. Time should be represented according to the biological question, such as age, follow-up duration, treatment exposure, BMI trajectory, or disease progression, and nonlinear patterns may require smoothing or state-space approaches.

In liver disease, repeated proteomic measurements are especially useful for evaluating therapeutic response. Semaglutide studies illustrate how treatment can shift MASH-related molecular programs and identify proteins associated with resolution [58,156]. For coordinated temporal shifts, multivariate repeated-measures methods, trajectory-oriented subgroups, or joint longitudinal-time-to-event models may be appropriate, but they require sufficient sample size, visit density, and validation [157,158,159].

### 7.6. Pathway and Network Analyses

Pathway and network analyses help organize differential protein lists into biological programs relevant to the overlapping obesity, T2D, and steatotic liver disease landscape. Across metabolic proteomics, enrichment commonly highlights inflammation, ECM remodeling, mitochondrial dysfunction, insulin signaling, lipid handling, and inter-organ communication using resources such as Reactome, Gene Ontology, Kyoto Encyclopedia of Genes and Genomes (KEGG), Search Tool for the Retrieval of Interacting Genes/Proteins (STRING), and Cytoscape [160,161,162,163].

Enrichment is a useful first pass but depends on the input protein list, background set, database, and statistical method. Network analysis adds value by identifying coordinated modules and hub proteins, including protein–metabolite or weighted gene co-expression network analysis (WGCNA) modules linked to adipose dysfunction, insulin resistance, depot-specific biology, and hepatic steatosis [28,58,60,162,164,165,200]. For proteomics, the measured protein set—not the whole genome—should generally be used as the enrichment background to reduce biased significance.

Over-representation analysis, rank-based enrichment, co-abundance networks, and protein interaction networks answer different questions and should not be treated as interchangeable. Over-representation analysis is readily interpretable but requires a threshold-defined protein list, making the result sensitive to the significance cutoff used for inclusion [201]. Rank-based enrichment retains information from all measured proteins and may detect coordinated but modest abundance changes, although results depend on the ranking statistic, gene set size, and correlation structure [202,203]. WGCNA and related approaches can identify cohort-specific co-abundance modules and module–phenotype relationships, but their stability depends on sample size and correlation structure. Because these networks are correlation-based, they do not establish physical interactions, directionality, or causality [28]. STRING integrates curated, experimentally supported, predicted, and text-mined protein associations, whereas Cytoscape provides an environment for importing, integrating, analyzing, and visualizing molecular interaction networks [204]. STRING integrates curated, experimentally supported, predicted, and text-mined protein associations, whereas Cytoscape provides an environment for importing, integrating, analyzing, and visualizing molecular interaction networks [205].

For enrichment analyses, the reference universe should comprise proteins that passed QC and were measurable on the relevant platform and in the relevant tissue, rather than the entire genome [206]. In paired tissue–plasma studies, each compartment should first be analyzed according to its own abundance distribution, missingness structure, and biological context before concordance is assessed [41].

PTM datasets require site-level or PTM signature analysis, rather than relying exclusively on conventional protein-level pathway mapping [207]. Phosphosite changes should be interpreted in relation to parent protein abundance, site localization confidence and the quality of kinase–substrate annotations [208]. Mapping modified peptides only to total-protein pathways can obscure whether a signal reflects altered protein abundance or an altered regulatory state [207]. Kinase–substrate enrichment analysis and PTM-specific signature enrichment may provide greater mechanistic resolution [207,209], but incomplete substrate annotation and ambiguous site assignment should be acknowledged [205,208].

Network outputs should not be interpreted as causal by default. Hub proteins may reflect annotation density or technical connectivity, so pathway and network results are most convincing when supported by study design, tissue context, longitudinal change, multi-omics evidence, or external validation [58,60,80,164,166].

### 7.7. Feature Selection and Validation for Biomarker Panels

Feature selection translates high-dimensional proteomic profiles into compact, clinically actionable panels capable of differentiating the overlapping phenotypes of obesity, T2D, and steatotic liver disease. Because metabolic proteins exhibit high degrees of multi-organ crosstalk and strong collinearity, particularly circulating adipokines and hepatokines, and sample sizes are often modest, selected panels should be parsimonious, interpretable, measurable, biologically coherent, and validated beyond the discovery cohort [25,167,168,169,170].

Standard filter and wrapper methods often struggle to resolve this highly correlated metabolic noise. Consequently, embedded approaches, particularly regularized regression models like LASSO, elastic net, or advanced Bayesian variable selection methods, may be useful. In the context of the MASLD/MetALD landscape, these penalized approaches can shrink the coefficients of generic inflammatory markers, allowing the sparse, disease-specific proteomic features driving hepatic injury to emerge [167,169,171,210].

Validation is the decisive step. Feature selection and tuning should occur inside training or inner resampling loops to avoid information leakage, and final panels should be evaluated for discrimination, calibration, clinical utility, external validity, platform portability, and biological plausibility [25,40,41,167,170,171,172,173,174,175,176,179,210,211,212,213].

### 7.8. pQTL, Colocalization, and MR

pQTL mapping, colocalization, and MR help distinguish proteins that may participate in disease mechanisms from proteins that are only correlated with clinical states. pQTLs provide genetic anchors for circulating proteins, while colocalization tests whether protein and disease associations at a locus are consistent with the same causal signal [25,177,178,179,180].

MR then uses genetic instruments to test whether genetically predicted protein abundance is consistent with disease causation, rather than downstream correlation. These approaches have prioritized proteins in obesity, visceral adiposity, T2D, MASLD, fibrosis, and related metabolic traits [177,179,180,181,182,214,215].

Genetic analyses remain sensitive to horizontal pleiotropy, linkage disequilibrium, weak instruments, isoform or assay specificity, and ancestry differences [216]. They are most useful when integrated with tissue relevance, longitudinal change, platform validation, and biological plausibility [25,181,182,217,218,219,220,221,222,223,224,225,226].

### 7.9. Complementary Multi-Omics Integration with Transcriptomics and Metabolomics

This subsection separates complementary omics integration from the core proteomics workflow. Transcriptomic, metabolomic, lipidomic, clinical, and genetic data are informative but are not interchangeable with protein measurements. Proteomics provides a direct readout of effector biology, but single-omics analysis can remain descriptive. Integrating transcriptomics, metabolomics, clinical phenotypes, and genetics can connect tissue remodeling, circulating proteins, metabolic flux, and therapeutic targets across the obesity–T2D–liver axis [80,164,183,184,185].

Modern integration increasingly moves beyond late-stage overlap of separate omics results toward intermediate matrix factorization, latent-factor models, network reconstruction, and graph-based approaches. These methods can reveal shared programs that are not apparent from any single data layer [227,228,229]. Their added complexity also increases risks from scale differences, missingness, batch effects, and overfitting; independent or held-out validation is therefore essential.

Multimodal networks are particularly valuable for resolving tissue–plasma discordance. By linking adipose or hepatic pathology with plasma hepatokines, adipokines, EV proteins, metabolites, and genetic evidence, they can prioritize biomarkers that are both accessible and mechanistically interpretable [28,40,41,42,185,186,187,188,189,190,230,231].

### 7.10. Benchmarking and Reproducibility

As metabolic proteomics scales, analytical flexibility in preprocessing, missing-data handling, normalization, batch correction, covariate adjustment, and feature selection can change the set of significant proteins obtained from the same dataset. Benchmarking is therefore part of scientific inference, not a purely technical exercise [30,142].

Formal benchmarking should compare complete pipelines using experimental controls, cross-cohort replication, or simulations with known ground truth. Reproducibility also requires complete reporting of software versions, parameters, preprocessing choices, model specifications, and code, along with data deposition through resources such as ProteomeXchange and containerized workflows when possible [232,233,234].

## 8. Limitations and Translational Perspectives

### 8.1. Current Limitations, Knowledge Gaps, and Practical Priorities

Current metabolic disease proteomics is limited by cross-sectional designs, inconsistent phenotyping, preanalytical and platform heterogeneity, tissue–plasma discordance, incomplete proteoform resolution, limited ancestral and clinical diversity, and insufficient external validation. Proteoform-level heterogeneity is particularly difficult to capture in large-cohort bottom–up workflows. Top–down approaches can resolve intact proteoforms and reveal cellular heterogeneity that is obscured by protein-level aggregation, although complementary bottom–up and top–down strategies are currently needed to balance proteome depth, throughput, and proteoform resolution [235,236]. Practical priorities include prespecified analysis plans, standardized collection and reporting, explicit modeling of missingness and covariates, external or held-out validation, orthogonal assay confirmation, paired tissue or genetic support, and evaluation of whether a proposed endotype improves a clinical decision, rather than only a discrimination metric.

### 8.2. Translational Outlook and Future Directions

Viewing obesity, T2D, MASLD/MASH, and MetALD as an overlapping metabolic dysfunction spectrum provides a systems-level framework for stratification without implying a fixed disease sequence. Proteomics is central because it captures functional molecular outputs that mediate inter-organ communication, but translation requires stronger longitudinal study designs. Most studies remain cross-sectional, limiting causal inference and the characterization of disease trajectories. Future research should prioritize repeated sampling and advanced longitudinal approaches, including mixed-effects, state-space, stochastic-process, or Gaussian-process models to better capture progression and transitions between disease states [237,238].

Phenotyping must also improve, especially for metabolic liver disease. Although MASLD terminology has replaced nonalcoholic fatty liver disease (NAFLD) in many contexts, separating metabolic and alcohol-related contributions remains difficult [4]. Reliance on self-reported alcohol intake introduces substantial misclassification bias. Incorporating objective biomarkers such as PEth and ethyl glucuronide could improve disease classification, strengthen proteomic signal detection, and facilitate more precise endotyping across the MASLD–MetALD spectrum [239].

Clinical translation further depends on cross-platform harmonization and rigorous causal prioritization. MS discovery findings may not transfer directly to targeted assays, highlighting the need for standardized reference materials, inter-laboratory benchmarking, assay harmonization, and transparent reporting standards [78]. Integrating proteomics with genetic evidence through MR, pQTLs, and colocalization can help prioritize proteins that are more likely to be causal drivers, rather than downstream correlates, of disease [240,241,242].

A further priority is proteoform-aware endotyping. Most large-cohort bottom–up MS and affinity studies quantify peptides, protein groups, or assay targets, rather than individual proteoforms. Functionally distinct species generated by alternative splicing, sequence variation, proteolysis, or PTMs may therefore be collapsed into one protein-level estimate. Future studies should combine proteoform-resolving approaches, including top–down, middle–-down, glycoproteomic, and phosphoproteomic workflows, with computational models that represent this ambiguity, while recognizing the current trade-off between molecular resolution and cohort-scale throughput.

Ultimately, metabolic proteomics should support clinically actionable endotypes that guide prevention, prognosis, treatment selection, and monitoring. Achieving this goal will require reproducible pipelines, longitudinal data, precise phenotyping, diverse cohorts, cross-platform validation, proteoform-aware assay design, and evidence that proteomic endotypes improve decisions in real-world care. These priorities are intended as practical recommendations for moving metabolic proteomics from literature summaries to reproducible, mechanism-informed endotyping.

## Figures and Tables

**Table 1:** Proteomic platforms and design considerations across the metabolic dysfunction spectrum.

Approach	Best Applications	Key Strengths, Limitations, and Validation Needs	Citations
Plasma/serum proteomics	Large cohorts, repeated sampling, biomarker discovery, and risk stratification.	Accessible and scalable; limited by dynamic range, high-abundance proteins, preanalytical variation, and uncertain tissue origin. Validate high-priority candidates with paired tissue data or an orthogonal assay.	[36,37,38,39,40,41,42]
Tissue proteomics	Mechanistic studies of adipose, liver, muscle, pancreas, or disease-stage biology.	Reveals local pathology and tissue context; limited by invasive sampling, tissue heterogeneity, and small cohorts. Standardized collection and paired plasma analysis improve translation.	[40,41,42,43,44,45,46]
MS discovery (DDA/DIA)	Protein identification, broad discovery, and reproducible quantification in tissue or plasma cohorts.	DDA supports exploratory depth but has more stochastic missingness; DIA improves completeness but depends on acquisition and software choices. Confirm priority candidates by targeted MS.	[36,47,48,49,50,51,52]
Affinity platforms	High-throughput plasma profiling in epidemiologic, longitudinal, and proteogenomic studies.	Olink and SomaScan enable high throughput and small sample volumes; target recognition and cross-platform concordance are incomplete. Confirm high-priority findings by MS or an independent affinity assay.	[25,53,54,55,56,57,58]
Targeted and post-translational modification PTM-focused proteomics	Verification of candidate panels and mechanistic interrogation of signaling or protein regulation.	Targeted MS supports precise predefined panels; PTM workflows reveal signaling states but require enrichment, site localization, and reference standards.	[5,36,40,59,60]
Secretome, EV, single-cell, and spatial proteomics	Inter-organ crosstalk, vesicle cargo, cell-state heterogeneity, and tissue microenvironments.	Highly informative for mechanism and cellular context; sensitive to isolation, sample preparation, and throughput limits. Standardized protocols and independent validation are essential.	[61,62,63,64,65,66,67,68,69]

Abbreviations: MS, mass spectrometry; DDA, data-dependent acquisition; DIA, data-independent acquisition; PTM, post-translational modification; EV, extracellular vesicle.

**Table 2:** Shared biological pathways and inter-organ signals across MASLD/MASH, MetALD, obesity, and T2D.

Process or Axis	Main Biological Signal	Proteomic/Endotyping Relevance	Citations
Inflammation	Adipose macrophage recruitment, cytokine signaling, Kupffer-cell activation, and hepatocyte stress.	Defines inflammatory burden and helps distinguish steatosis from progressive MASH-like injury.	[110,111]
Mitochondrial dysfunction and oxidative stress	Fatty acid overload, incomplete beta-oxidation, reactive oxygen species, and impaired metabolic flexibility.	Connects tissue energy stress with systemic insulin resistance and liver injury.	[112,113,114]
ECM remodeling and fibrosis	Hepatic stellate-cell activation, collagen deposition, matrix remodeling, and fibrosis progression.	Identifies advanced injury biology and fibrosis-linked biomarker candidates.	[115,116,117]
Proteostasis and cellular stress	ER stress, unfolded protein response, autophagy disruption, and stress-response proteins.	Captures cellular injury programs that may persist even when abundance changes are modest.	[118,119]
Lipid handling and lipotoxicity	Free fatty acid flux, de novo lipogenesis, toxic lipid species, and altered lipid transport.	Links obesity and insulin resistance to steatosis, beta-cell dysfunction, and progressive liver injury.	[97]
Complement, coagulation, and hemostasis	Complement activation, fibrinogen/plasminogen changes, and coagulation-related remodeling.	Places inflammatory amplification and fibrosis within a systemic plasma protein signature.	[120,121]
Inter-organ crosstalk and EV signaling	Adipokines, hepatokines, myokines, pancreatic stress signals, circulating EV cargo, and treatment-responsive proteins.	Explains why plasma proteomics can monitor risk, progression, and therapeutic response across organs.	[41,42,58,110,122,123,124,125]

Abbreviations: MASH, metabolic dysfunction-associated steatohepatitis; MASLD, metabolic dysfunction-associated steatotic liver disease; MetALD, metabolic dysfunction-associated alcohol-related liver disease; T2D, type 2 diabetes; ECM, extracellular matrix; ER, endoplasmic reticulum; EV, extracellular vesicle.

**Table 3:** Bioinformatics and biostatistical framework for proteome-informed endotyping.

Analytical Step	Purpose	Best-Practice Emphasis	Citations
Preprocessing and QC	Convert raw spectra or assay outputs into reliable quantitative protein matrices.	Perform tissue- and platform-aware QC; track peptide-to-protein inference and provenance; report run-, sample-, and feature-level diagnostics.	[130,131,132,133,134]
Missing-data handling	Distinguish technical missingness from abundance-dependent censoring and informative absence.	Quantify missingness by tissue, group, protein, and sample; align imputation or model-based inference with the acquisition-specific missingness mechanism.	[30,135,136,137,138,139,140]
Normalization and batch correction	Improve comparability while preserving biological structure.	Normalize within homogeneous tissues or platforms; apply correction only when diagnostics show reduced technical variation without erasing biology.	[130,135,141,142,143,144]
Differential abundance and covariates	Estimate protein–disease associations while adjusting for confounders.	Use design-aware linear or mixed models; include age, sex, adiposity, medication, kidney/liver function, and disease stage when appropriate.	[139,145,146,147,148,149,150,151,152,153]
Longitudinal modeling	Capture within-person trajectories, treatment response, and temporal disease dynamics.	Prefer mixed-effects models as a starting point; match time scale to biology and consider multivariate or joint models for complex trajectories.	[56,58,154,155,156,157,158,159]
Pathway and network analysis	Move from protein lists to coordinated biological programs.	Use the measured proteome as the enrichment background; validate network modules and hub proteins with tissue context, longitudinal evidence, or orthogonal omics.	[28,80,160,161,162,163,164,165,166]
Feature selection and validation	Translate high-dimensional profiles into compact biomarker panels.	Keep feature selection and tuning inside resampling; prioritize parsimony, calibration, external validation, platform portability, and biological plausibility.	[25,40,167,168,169,170,171,172,173,174,175,176]
Genetic and multi-omics integration	Prioritize proteins that are mechanistically interpretable and not only associative.	Separate core proteomics analysis from complementary omics integration; use pQTLs, colocalization, MR, and other layers to connect circulating markers with tissue biology and causality.	[40,41,42,80,164,177,178,179,180,181,182,183,184,185,186,187,188,189,190]

Abbreviations: QC, quality control; pQTL, protein quantitative trait locus; MR, Mendelian randomization.

## Data Availability

No new data were created or analyzed in this study. Data sharing is not applicable to this article.
